# HLA allele distributions and associations in a cohort of LTNPs from China

**DOI:** 10.1186/1742-4690-8-S1-A84

**Published:** 2011-06-06

**Authors:** Mohammad A Rai, Stella Chun Hao, Marie-Eve Blais, T Rostron, Y Zhang, KY Xu, H Yan, Andrew J McMichael, T Dong, Sarah Rowland-Jones

**Affiliations:** 1 MRC Human Immunology Unit, Weatherall Institute of Molecular Medicine, University of Oxford, OX3 9DS, UK; 2 Beijing You An Hospital, Capital Medical University, Beijing, PRC

## Background

Various studies have shown that HLA polymorphisms are associated with susceptibility/resistance to HIV-1 infection, and therefore influence the rate of disease progression of HIV/AIDS in individuals of different ethnic backgrounds. Association of HLA with HIV/AIDS has been studied extensively within Caucasians and Africans. However, limited data are available from China.

## Methodology

HIV Positive Samples were collected from the SM village in Henan province: SM Cohort. These subjects got infected in the early 90s after taking part in an illegal plasma donation scheme that became contaminated by HIV-1 infection. Our data show that they were infected by a single or a few closely related clade B strains. Control samples collected from a neighboring village. DNA was extracted, HLA-typing done and HLA analysis computed using Arlequin and SAS.

## Results

Genetic profile of the cohorts was obtained. On 1 locus: analysis: HLA A*02 (P=0.35), HLA B*15(P=0.02) and HLA B*44(P=0.02) were found to be statistically significant. 2 loci differences include B*13 C*06, B*13 C*06, A*02 C*07, A*24 C*03. 3 loci: A*30 B*13 DRB1*07. Similarly, viral loads and CD4 count associations were computed to find associations co-relating with the HLAs.

## Conclusions

This is the first analysis for HLA distribution amongst LTNPs from China, inferring their HLA co-relates with a delayed progression to HIV/AIDS. In our cohort, for slow and long-term non-progressors, there is an enrichment of specific HLAs which appear to be associated with delayed disease progression. Next plan is to initiate T Cell work and also correlate KIR/NK cell responses.

